# Supplementary material for: SyNDI: synchronous network data integration framework
Source: BMC Bioinformatics. 2018 Nov 6;19:403. doi: 10.1186/s12859-018-2426-5 (PMC6219086; doi:10.1186/s12859-018-2426-5)
Supplement: Supplementary file 5 — Files of the example presented in Additional file 4. This additional file is a zip package that contains all files used in Additional file 4. Detailed descriptions of each of these files are presented in a README file included in this zip package. (GZ 30411 kb) [file 12859_2018_2426_MOESM5_ESM.gz › SyNDITools/Meme2Fimo/meme_4.9.0_4.tar.gz!/meme_4.9.0/doc/miao.xml]

MIAO - a motif search tool


# MIAO -- not currently supported

**Usage:**

`miao [options] <motifs> <database>`

**Description:**

`miao`
(**m**otifs **i**n **a**ny **o**rder)
Searches a sequence database for clusters of known motifs.
As in `beadstring`
motifs are represented using a hidden Markov model,
but `miao` uses the complete or star topologies.
This allows the motifs to appear in any order.
A full description of the algorithm is found in:

Grundy. *A Bayesian Approach to Motif-based Protein
Modeling*. Ph.D. dissertation. University of California, San
Diego. June, 1998.

**Input:**

- `<motifs>` is a list of motifs, in MEME format.
- `<database>` is a collection of sequences in
  FASTA format.

**Output:**

An XML file using the
CisML
schema.

**Options:**
